# The FOCUS model: A guide to promote patient partnership in clinical trial designs

**DOI:** 10.1017/cts.2026.10719

**Published:** 2026-03-09

**Authors:** Jennifer Catena Davis, Patrick Corr, Amy Raymond, Sabrina Figueiredo

**Affiliations:** 1 School of Medicine and Health Sciences, The George Washington University, USA; 2 Worldwide Clinical Trials, USA

**Keywords:** Patient-researcher partnerships, clinical trials, Duchenne muscular dystrophy, rare disease, clinical drug development

## Abstract

**Background:**

Evidence suggests that partnerships between researchers and patient communities result in clinical trials that better reflect the patient experience, but there are few documented and successful models of such partnerships. Within the clinical research landscape, the Duchenne Muscular Dystrophy (DMD) community has emerged as a positive outlier in this regard, having engaged in a research partnership over multiple years and in the approval of several disease-modifying therapies where there were previously none.

**Methodology:**

The successful partnership factors observed in the DMD community were mapped onto the three levels of the Behavior Change Wheel framework. This mapping created an academic model of patient community readiness for research partnerships. The academic model was further translated into a user-friendly, pragmatic model and blueprint.

**Results:**

The FOCUS Model outlines the development of a research partnership from the ground up, where each theme represents an essential component of the partnership structure. Accompanying this model is a discussion guide that communities can use in meetings or workshops to assess and enhance their readiness for research partnerships.

**Conclusions:**

The FOCUS model and its blueprint offer generalizable approaches that other rare disease patient communities can leverage to foster effective and lasting partnerships with scientific, medical, and regulatory stakeholders. The approach proposed in this study has the potential to help both the clinical research community and rare disease patient communities overcome typical barriers to rare disease clinical development.

## Introduction

In modern times novel medical treatments become available for public use after systematic evaluation in a series of adequately designed, well-controlled clinical trials [1]. In most cases, in addition to a favorable safety profile, clinical trials of new drugs must show robust evidence of clinical benefit; the drug must improve the way the patient feels, functions, or survives [1–3]. While measuring survival is typically straightforward, feeling and function often comprise constructs that are difficult to measure and may be known only to the patient; therefore, choosing a meaningful clinical endpoint by which a drug’s efficacy can be evaluated is of paramount importance. Unfortunately, clinical trials often fail to measure outcomes that are meaningful to patients, leading to inconclusive trial results, regulatory conflict, loss of confidence in the drug development system, and poor prediction of product performance in the general population [4]. To meet the scientific and ethical imperative of designing clinical trials in a way that will further the goal of improving patients’ lives and public health, trials must measure outcomes that patients find meaningful [5–7].

The design of clinical trials often presents significant obstacles to patient participation, even as they fall short in measuring patient-centered endpoints. Patient participation in clinical trials is essential to the development of safe and effective medical treatments; however, the design of clinical trial protocols may require patients to devote significant time, undergo substantial travel, incur expenses, and endure numerous invasive and time-consuming assessments, which can be significantly burdensome for study participants [8,9]. Clinical researchers have a moral and ethical imperative to minimize patient burden associated with clinical trial participation; however, excessive patient burden can have a business cost as well, resulting in patient recruitment and retention problems, exclusion of an appropriately diverse patient population, inability of a sufficient number of patients to complete endpoint assessments, threats to internal and external validity, and other operational risk [9,10]. Patient burden may be reduced through the use of patient-centered clinical trial design features, but optimization of patient-centered clinical trial design requires the proactive assessment of patient burden associated with clinical trial protocols, something not often undertaken in a systematic fashion [10,11].

Researchers and regulators have begun to recognize the importance of partnerships with patient communities to improve trial design and research methods [5,12–15]. Evidence suggests that greater patient input into the design of clinical trials can improve the evaluation of risks and benefits of new drugs by ensuring that this analysis reflects outcomes and values that are important to patients. For instance, patient advocacy organizations, such as those involved in spinal muscular atrophy research, have shown that early patient involvement can accelerate access to treatments and improve understanding of a product’s value, thus enhancing recruitment efforts [16,17]. The RECOVAC project demonstrated that patient engagement could influence research conduct and outcomes, highlighting the importance of clear communication [18].The InMe trial incorporated Patient and Public Involvement (PPI) to co-design a therapeutic model, resulting in comprehensive study procedures that were feasible and acceptable to participants [19]. The APPETIZE study highlighted the positive impact of including patient representatives in trial design, which led to enhanced trial protocols and the generation of high-quality, patient-relevant data [20]. Despite the existence of various models and frameworks for patient involvement [21], the literature indicates a lack of standardized methods for partnering with patients in trial design [22–24].

An exemplary case of patient partnership in clinical trial design comes from the Duchenne Muscular Dystrophy (DMD) community, who worked effectively with researchers and regulators toward optimizing clinical trial endpoint selection and codifying their knowledge in the first patient advocacy-initiated FDA guidance document [25–32], in addition to five disease-modifying treatments being approved by the FDA within the last six years [33]. This is an extraordinary accomplishment and one that many other patient and clinical research communities would like to achieve.

To address this gap, this study will explore the following research question: What factors that contributed to the success of the DMD research partnership should be included in a model intended to help other patient communities create similarly successful partnerships? By answering this question, this study aims to develop a model for successful partnerships between patient communities and clinical researchers to improve the design of future clinical trials.

## Material and methods

The patient partnership model was developed by triangulating the thematic findings from a qualitative descriptive case study described elsewhere [34]. The parent study aimed to identify the factors impacting the ability of the DMD community to form and sustain a successful partnership with industry researchers to drive development of new DMD therapies. Individuals who participated in the study were required to have been involved in any aspect of industry-sponsored clinical trial design for DMD and to be over the age of 18. Participants were people with DMD (inclusive of Becker Muscular Dystrophy [BMD], a closely related disorder), caregivers, researchers, and members of patient advocacy communities [34].

The Behavior Change Wheel (BCW), a determinant framework describing factors impacting behavior change [35], guided the interpretation of the findings. At the center of the BCW is the Capability Opportunity Motivation – Behavior (COM-B) Model. For a given behavior to take place, the people concerned must have the capability to do it, meaning they must have knowledge, skills, etc. to perform the behavior. They must also have the opportunity to do so, meaning there must be a conducive physical and social environment for the behavior to occur. Finally, they must have the motivation to carry out the behavior, meaning they must be more highly motivated to engage in the desired behavior than to not do so or to perform a competing behavior [35,36].

As an implementation science model, the BCW is designed to inform interventions that will lead to the execution of a desired behavior. The inner wheel of the BCW identifies nine intervention functions, or components of a behavioral intervention, that are expected to exert influence on the desired behavior: education, persuasion, incentivization, coercion, training, restriction, environmental restructuring, modeling, and enablement. The outer wheel of the BCW identifies seven policy categories that an intervention may seek to influence in order to support the delivery of the relevant intervention functions: communication/marketing, guidelines, fiscal measures, regulation, legislation, environmental/social planning, and service provision. The components of the BCW are defined and described in detail in “The Behaviour Change Wheel: A Guide to Designing Interventions,” by Susan Michie, Lou Atkins, and Robert West [36]. In this study, the desired behavior to be achieved is for patient communities to engage in partnerships to improve the design of clinical trials.

The 15 factors of successful partnership identified by Davis and colleagues [34] were mapped to the intervention functions and policy categories from the BCW, which in turn correlate to core behavior determinants of the BCW model, namely capability, opportunity, and motivation.

## Results

Factors of successful partnerships between researchers and patient communities described elsewhere informed the partnership model proposed here. By mapping factors of successful partnerships [34] to the three levels of the BCW framework [36] we developed a model to facilitate the implementation of partnerships between patient communities and researchers.

Table 1 maps the factors of successful partnership in the DMD community to their relevant intervention functions, which are in turn mapped to the policy categories that are expected to support them. The connections between intervention functions and policy categories are defined by the authors of the BCW model [36]. The BCW offers a comprehensive model for understanding and designing interventions that target behavior change by systematically aligning determinants, intervention functions, and policy categories. At the highest level, the first column groups the determinants of successful partnership into overarching themes – including **F**ighting for Future Generations, **Fo**stering a Culture of Collaboration, **C**reating a Cohesive Community that Leverages its Resources, **U**tilizing Legislative Advocacy to Influence the Regulatory Climate, *and the* **S**pecific Nature of DMD (FOCUS). *Each theme encompasses several factors (second column) that represent partnership success, such as characteristics of the condition, funding opportunities, providing patient education, convening stakeholders, and maintaining consistent activity. For each of these factors, the third column includes relevant intervention functions, which represent broad categories of strategies that can be employed to influence* behavior. Consistent with the BCW taxonomy, these include education, training, persuasion, incentivization, coercion, environmental restructuring, modeling, and enablement. For example, factors such as “convene stakeholders” or “provide patient education” are primarily associated with education, persuasion, environmental restructuring, and modeling. Conversely, “funding opportunities” or “regulatory opportunities” align more strongly with incentivization, persuasion, coercion, and enablement. Each intervention function is then connected to one policy category (fourth column), which represents the higher-level mechanisms that support, scale, and sustain interventions. These policy levers – communication and marketing, guidelines, fiscal measures, regulation, environmental and social planning, and service provision – are derived directly from the BCW framework and provide a practical bridge between intervention design and system-level implementation. For instance, communication and marketing policies may facilitate awareness and advocacy initiatives across the DMD community (which influences the characteristics of the condition); on the other hand, service provision policies can enable the sustained delivery of educational, clinical, and psychosocial support programs. Finally, the fifth column introduces the COM-B components most likely to be influenced by the intervention functions (third column). For instance, education and training functions predominantly enhance psychological capability, enabling individuals and organizations to develop the skills and understanding necessary for effective participation. Modeling and persuasion contribute to reflective and automatic motivation, reinforcing shared purpose and sustained engagement. Meanwhile, environmental restructuring and enablement influence physical and social opportunities by improving access to resources, communication infrastructure, and partnership networks.

**Table 1.** Mapping between factors of successful partnership and the domains of the behavior change wheel

Themes	Factors of successful partnership	Intervention functions	Policy categories	COM-B
Specific nature of DMD	Size of community	Modeling Enablement	Communication/marketing Service provision	Psychological capability Reflective Motivation Physical Opportunity Social Opportunity
	Characteristics of condition	Persuasion	Communication/marketing	Reflective Motivation Physical Opportunity
Utilize legislative advocacy to influence funding and regulatory climate	Funding opportunities	Incentivization Enablement	Guidelines Fiscal measures Regulation Legislation	Psychological capability Reflective Motivation Automatic motivation Physical Opportunity Social Opportunity
	Regulatory opportunities	Persuasion Incentivization Coercion Enablement		
Create a cohesive community and leverage its resources	Provide patient education	Education	Communication/marketing Service provision	Psychological capability Reflective Motivation
	Leverage internal expertise	Education Training Modeling Enablement		Physical capability Psychological capability Reflective motivation Automatic motivation Physical opportunity Social opportunity
	Provide family services	Education Incentivization Enablement	Communication/marketing Service provision Environmental/social planning	Physical capability Reflective motivation Automatic motivation Physical opportunity Social opportunity
	Connect families to each other	Education Incentivization Modeling Enablement		
	Organize for influence	Training Modeling Enablement	Communication/marketing Service provision Environmental/social planning Guidelines	Physical capability Psychological capability Reflective motivation Automatic motivation Physical opportunity Social opportunity
Foster a culture of collaboration	Convene stakeholders	Education Persuasion Environmental Restructuring Modeling	Communication/marketing Service provision Environmental/social planning Guidelines	Psychological capability Reflective motivation Automatic motivation Physical opportunity Social opportunity
	Create collaboration opportunities	Environmental Restructuring Modeling		
	Create precompetitive collaboration environment	Education Incentivization Environmental Restructuring Modeling Enablement	Communication/marketing Service provision Environmental/social planning Guidelines Regulation	
Fighting for future generations	Maintain consistent activity	Persuasion Environmental Restructuring Modeling	Communication/marketing Service provision Environmental/social planning Guidelines	Psychological capability Reflective motivation Automatic motivation Physical opportunity Social opportunity
	Work for the good of the whole	Environmental Restructuring Modeling Enablement		
	Long-term strategy	Environmental Restructuring Modeling	Communication/marketing, Service provision Environmental/social planning Guidelines Regulation	

**Figure 1.**
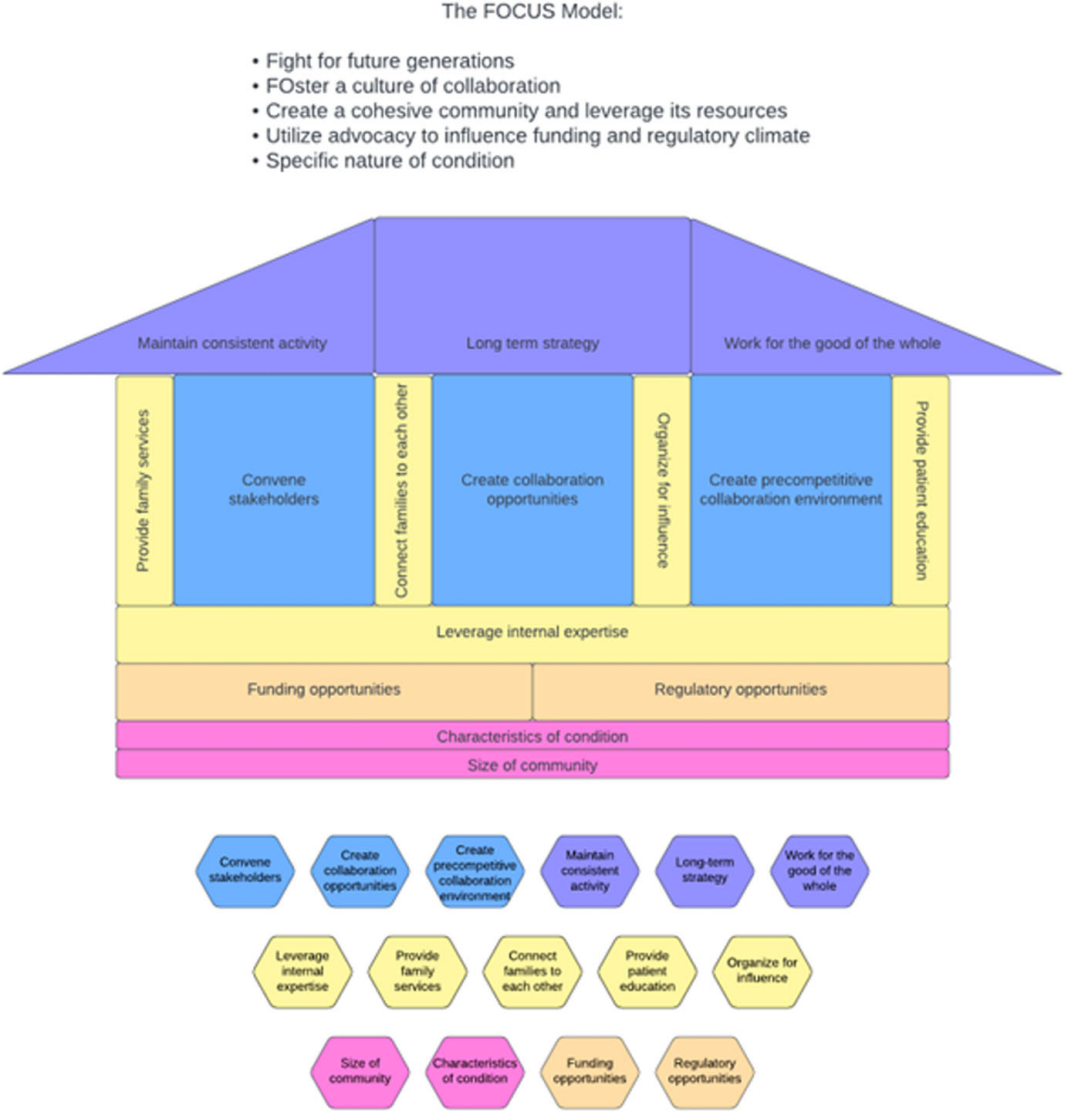
The FOCUS model – a user-friendly model for patient communities.

The mapping of successful partnership factors to the COM-B allow for the identification of specific interventions to drive change (i.e., form a partnership). To support knowledge translation and dissemination, findings from Table 1 – appropriate for an academic environment – were organized into a pragmatic model (Figure 1) and a blueprint (Tables 2–6) to facilitate its adoption by patient communities and applicable to real-world research partnerships. The proposed model retains the themes and successful partnership factors identified in Table 1 but reorganizes them into the form of a physical structure that helps to explain the role each theme plays in constructing and strengthening a research partnership. Each theme becomes a key component of the structure, from the ground to the roof. On the other hand, intervention functions and COM-B components inform the blueprint presented in Tables 2–6.

**Table 2.** The FOCUS MODEL blueprint for guiding the implementation of patient partnerships: The ground

	The ground – specific nature of the condition
**Relevant partnership factors**	Size of community Characteristics of condition
**Attributes of successful partnership factors**	The specific nature of the condition a community faces is the ground upon which the rest of the house is built. The size of the patient community determines the extent of resources and expertise that will be found within the community itself, which impact the volume of work the community can undertake and the ease with which they can do so. The size of the community also corresponds to the size of the market that will ultimately be available when treatments are marketed and can impact the prioritization of the disease for public funding. The characteristics of the condition, including its severity and prevalence, also impact prioritization for funding and may influence how attractive the condition is perceived to be as a research target.
**Questions to consider**	**Size of community** How many families are impacted by this disease? Are these families geographically distributed or disproportionately located in specific areas? Is our community big enough to act on our own, or should we join with other communities (i.e., other communities with similar conditions) for a bigger impact? **Characteristics of condition** How well do we understand the medical science behind our condition? How well does the research community understand the medical science behind our condition? Are people outside our community aware of our condition? How do they perceive it? Is that perception accurate? What challenges do families living with this condition face?
**Possible actions**	To improve in this area, consider interventions that provide role modeling, persuade others through communication initiatives, or enable the community by increasing capacity or opportunity.

**Table 3.** The FOCUS MODEL blueprint for guiding the implementation of patient partnerships: The foundation

	The foundation – utilize advocacy to influence funding and regulatory climate
**Relevant partnership factors**	Funding opportunities Regulatory opportunities
**Attributes of successful partnership factors**	Optimizing the funding and regulatory environment for a particular condition provides a solid foundation for research partnership activities. Some patient groups have made important gains by using legislative advocacy to create a funding and regulatory climate favorable to their community. Groups may consider advocating for disease-specific funding through targeted legislation. A secure funding stream can greatly increase the volume of research that can be conducted for a particular condition and may make it a more attractive research target for researchers. Communities may also look into whether any existing legislation may enable dedicated funding for their condition. Legislative action, including the Patient Focused Drug Development (PFDD) provisions of PDUFA-5, has created regulatory incentives that enable the participation of patient communities in the drug development process. Communities may consider whether they would be good candidates for the PFDD process, or whether they could benefit from the development of disease-specific regulatory guidance.
**Questions to consider**	**Funding opportunities** How does my condition align with NIH funding priorities? Could my community benefit from disease-specific funding legislation? What kinds of advocacy assistance does my community need to increase the amount and stability of available funding for our condition? **Regulatory opportunities** What is our community’s relationship with the regulators? Does our community have access to PFDD meetings? What upcoming regulatory meetings would benefit from patient representation?
**Possible actions**	To improve in this area, consider interventions that persuade others through communication initiatives, enable the community by increasing capacity or opportunity, provide incentives for change, or coerce change through regulation.

**Table 4.** The FOCUS MODEL blueprint for guiding the implementation of patient partnerships: The pillars

	The pillars – create a culture of community and leverage its resources
**Relevant partnership factors**	Leverage internal expertise Provide family services Connect families to each other Provide patient education Organize for Influence
**Attributes of successful partnership factors**	These factors form the pillars that raise and support the structure of the partnership. The most foundational of these is leveraging internal expertise. Leveraging relevant expertise that already exists within the community, such as medical, nursing, business, legislative, or lobbying expertise, provides the community with insider knowledge about the often-complex systems that must be navigated to achieve their goals. Leveraging internal expertise allows a community to accomplish more with fewer external resources. To marshal these internal resources, patient groups will benefit from creating a community that is truly cohesive and gives families reasons to connect to the community, to stay connected, and ultimately to contribute their time, talent, and resources to enable the community to achieve their goals. To incentivize families to become connected a community should provide resources or services that families find valuable, bringing them to the table and demonstrating the value of community connection. Connecting families to each other creates a network of peer support that keep families involved and allows them to learn from each other’s experiences, maintaining connection and increasing the community’s capacity. The community structure forms an ideal framework within which to offer patient education, allowing families to better advocate for themselves when seeking care and equipping them with the information they need to become effective and credible research partners. Once this capacity is built within the community, the community is ready to organize for influence, reducing barriers for families to engage in advocacy and research opportunities and enabling the community to present a united front in these efforts.
**Questions to consider**	**Leverage internal expertise** What expertise do members of our community already possess? What expertise would be relevant to our community’s goals? Do we have medical, nursing, social work, business, marketing, communications, government, or lobbying expertise that might benefit our community? What connections do our community members have in the wider world? How might those connections benefit the community’s goals? **Provide family services** When families are newly diagnosed, what are their immediate concerns? How easy is it for families to find our organization? Are we prepared to be responsive to the needs of our families? Can we provide connections to medical, social, or financial services that families need? **Connect families to each other** What makes families feel connected to our community? How can we help families stay connected to the community? How can more experienced families help newly diagnosed families? What opportunities can we provide for families to learn from each other? What do families find valuable about being part of a patient community? **Provide patient education** How well does the average affected family understand this condition? What information do families need to ensure they receive quality care? What information do families need to understand and interpret research being done on our condition? What information do families need to effectively advocate for our community? How can we help families be educated consumers of information about their condition? **Organize for influence** What are our community’s top priorities? How can we make families aware of advocacy opportunities? How can we lower barriers to participation in advocacy efforts? How can we maximize the time and talent available in our community to amplify our efforts? What external assistance do we need for our advocacy to be effective?
**Possible actions**	To improve in this area, consider interventions that provide education or training opportunities, provide incentives for change, provide role modeling, or enable the community by increasing capacity or opportunity.

**Table 5.** The FOCUS MODEL blueprint for guiding the implementation of patient partnerships: The rooms

	The rooms
**Relevant partnership factors**	Convene interested parties Create collaboration opportunities Create precompetitive collaboration environment
**Attributes of successful partnership factors**	Once a strong structure has been raised, powerful collaboration can take place within that structure. Patient organizations are the natural conveners of interested parties in their research environment; therefore, patient groups play a key role in fostering a culture of collaboration that enables all research interested parties to work together to solve wicked problems. The first key factor in promoting a culture of collaboration is to convene relevant interested parties, such as patients and researchers, by providing opportunities for them to come together and meet each other in the same physical space, for example, through community conferences. Once interested parties have had the opportunity to encounter each other, patient groups can more easily create opportunities for direct collaboration, such as by facilitating multi interested parties’ workshops aimed at solving specific challenges (such as outcome measure development) and by lowering barriers to research collaboration with patients by providing a central point of contact for patient partners. Patient communities are also in the unique position to create precompetitive opportunities for drug developers to work together on issues of common concern, such as solving safety problems common to an entire class of drugs.
**Questions to consider**	**Convene interested parties** How well do researchers understand our patient experience? How well do we understand challenges researchers face in working on our condition? What opportunities can we provide for patient families and researchers to learn from each other? What opportunities can we provide for families to share their stories with researchers and regulators? **Create collaboration opportunities** What opportunities can we provide for researchers to work together? What outcome measures do researchers use to study our condition? Do they represent our experience, or do we need new ones? How can we help researchers overcome challenges they face in working on our condition? How can we align our efforts with other organizations working on common problems? **Create precompetitive collaboration environment** What research challenges are common in our condition? What solutions would benefit the research community as a whole? How can we incentivize drug development companies to work together on common problems?
**Possible actions**	To improve in this area, consider interventions that provide education opportunities, persuade others through communication initiatives, provide incentives for change, restructure the environment to facilitate change, provide role modeling, or enable the community by increasing capacity or opportunity.

**Table 6.** The FOCUS MODEL blueprint for guiding the implementation of patient partnerships: The roof

	The roof
**Relevant partnership factors**	Maintain consistent activity Long-term strategy Work for the good of the whole
**Attributes of successful partnership factors**	To effect durable change, community efforts must be consistent and sustained over a long period of time, rather than being seen as one-time advocacy activities, for example, engaging in legislative advocacy activities annually at the start of each legislative session. Communities must also carefully consider and adopt long-term goals, and adopt strategies that serve these goals, rather than expecting a quick fix. Similarly, to achieve maximum impact goals and strategies should be adopted to benefit the community as a whole rather than individual patients and families.
**Questions to consider**	**Maintain consistent activity** How can we align our advocacy efforts with relevant legislative timelines (for example, the start of the Congressional session, budget cycles, etc.)? What fundraising and advocacy efforts should be done on a recurring basis? What frequency is most appropriate for our key activities? Are there recurring events to which we could tie advocacy efforts (for example, having a benefit fundraising team participate in an annual marathon, maintaining a presence at annual community events, etc.)? **Long-term strategy** What is a realistic drug development timeline for our condition? What is the status of basic scientific research into our condition? What foundational activities need to take place to enable drug development for our condition (for example, collection of natural history data, development of appropriate outcome measures, etc.)? What are our immediate, intermediate, and long-term goals for our community? **Work for the good of the whole** How well do we understand the priorities of our community? What challenges are common to many families? How do we prioritize the advocacy efforts of the community as a whole? How can we motivate families to advocate for each other?
**Possible actions**	To improve in this area, consider interventions that provide education opportunities, persuade others through communication initiatives, restructure the environment to facilitate change, provide role modeling, or enable the community by increasing capacity or opportunity.

### The FOCUS model

FOCUS is an acronym of the five themes associated with successful partnerships between clinical researchers and patients’ communities, including **F**ighting for Future Generations, **Fo**stering a Culture of Collaboration, **C**reating a Cohesive Community that Leverages its Resources, **U**tilizing Legislative Advocacy to Influence the Regulatory Climate, and the **S**pecific Nature of DMD (FOCUS).

The intention of the FOCUS model is to describe the creation of a research partnership from the ground up, in which each theme forms an essential component of the construction of the partnership structure. The specific nature of the condition is the ground; utilize advocacy to influence the funding and regulatory climate is the foundation; create a cohesive community and leverage its resources forms the pillars; foster a culture of collaboration becomes the rooms; fight for future generations is the roof. These themes are described in greater detail elsewhere [34].

#### The ground: the specific nature of the condition

The specific nature of the condition a community faces is the ground upon which the rest of the house is built. The size of the patient community determines the extent of resources and expertise that will be found within the community itself, which impact the volume of work the community can undertake and the ease with which they can do so. The size of the community also corresponds to the size of the market that will ultimately be available when treatments are marketed; this may impact the availability of public and private funding and the amount of interest the disease receives from potential drug developers. The characteristics of the condition, including its severity and prevalence, can also impact prioritization for funding and may influence how attractive the condition is perceived to be as a research target.

#### The foundation: utilize advocacy to influence the funding and regulatory climate

Optimizing the funding and regulatory environment for a particular condition provides a solid foundation for research partnership activities. The DMD community has demonstrated that leveraging legislative advocacy increases opportunities for future progress by increasing the availability of research funding and opening the door to opportunities to engage with regulators when they are evaluating new treatments. Patient groups may consider advocating for disease-specific funding through targeted legislation. Communities may also investigate whether any existing legislation may enable dedicated funding for their condition. Legislative action, including the Patient Focused Drug Development (PFDD) provisions included in the fifth authorization of the Prescription Drug User Fee Act (PDUFA-5), has created regulatory incentives that enable the participation of patient communities in the drug development process [24,37,38]. While these opportunities have been discussed here in the context of the DMD community, these opportunities may also be leveraged by other patient groups. Communities may consider whether they would be good candidates for the PFDD process or whether they could benefit from the development of disease-specific regulatory guidance.

#### The pillars: create a cohesive community and leverage its resources

These factors form the pillars that raise and support the structure of the partnership. The most foundational of these is leveraging internal expertise. Leveraging relevant expertise that already exists within the community, such as medical, nursing, business, legislative, or lobbying expertise, provides the community with insider knowledge about the often-complex systems that must be navigated to achieve their goals. Leveraging internal expertise allows a community to accomplish more with fewer external resources. To marshal these internal resources, patient groups will benefit from creating a community that is truly cohesive and gives families reasons to connect to the community, to stay connected, and ultimately to contribute their time, talent, and resources to enable the community to achieve their goals. To incentivize families to become connected a community should provide resources or services that families find valuable, bringing them to the table and demonstrating the value of community connection. Connecting families to each other creates a network of peer support that keeps families involved and allows them to learn from each other’s experiences, maintaining connection and increasing the community’s capacity. The community structure forms an ideal framework within which to offer patient education, allowing families to better advocate for themselves when seeking care and equipping them with the information they need to become effective and credible research partners. Once this capacity is built within the community, the community is ready to organize for influence, reducing barriers for families to engage in advocacy and research opportunities and enabling the community to present a united front in these efforts.

#### The rooms: foster a culture of collaboration

Once a strong structure has been raised, powerful collaboration can take place within that structure. Patient organizations are the natural conveners of the interested parties in their research environment; therefore, patient groups play a key role in fostering a culture of collaboration that enables all parties to work together to solve wicked problems. The first key factor in promoting a culture of collaboration is to convene relevant parties, such as patients and researchers, by providing opportunities for them to come together and meet each other in the same physical space (e.g., through community conferences). Once interested parties have had the opportunity to encounter each other, patient groups can more easily create opportunities for direct collaboration. Potential actions could include facilitating multiparty workshops aimed at solving specific challenges (such as outcome measure development) or lowering barriers to research collaboration with patients by providing a central point of contact for patient partners. Patient communities are also in the unique position to create precompetitive opportunities for drug developers to work together on issues of common concern, such as solving safety problems common to an entire class of drugs.

#### The roof: fight for future generations

To effect durable change, community efforts must be consistent and sustained over a long period of time, rather than being seen as one-time advocacy activities. For example, community members may engage in legislative advocacy activities annually at the start of each legislative session. Communities must also carefully consider and adopt long-term goals and adopt strategies that serve these goals, acknowledging that this is a process that will take time. Similarly, to achieve maximum impact goals and strategies should be adopted to benefit the community rather than individual patients and families.

Tables 2–6 show a patient community blueprint to enable an immediate adoption of the FOCUS Model. The blueprint includes a brief narrative description of each theme, followed by a list of discussion questions related to each factor within the theme. Each thematic section concludes with a brief comment noting intervention functions that might help bolster the community’s readiness for research partnership in that area based on the BCW. The blueprint document (Tables 2–6) is designed to be ready for application in a community meeting or workshop and can be applied flexibly by communities across a wide spectrum of readiness for research partnership. While it provides general intervention functions that communities may wish to explore, the blueprint is not designed to prescribe specific interventions to promote patient community readiness. Patient communities are numerous and varied and would require interventions tailored to their specific circumstances [39].

## Discussion

### Summary of findings

The clinical research partnership established by the DMD community is notable for the remarkable results achieved over four decades of sustained collaboration and persistence on the part of all interested parties. This study introduced a patient-informed model based on the BCW theoretical framework, aimed at enhancing patient partnerships to improve clinical trials. The patient partnership model (FOCUS model) is accompanied by a workshop-ready blueprint that serves as a detailed and pragmatic guide, including a series of discussion questions for communities to use to assess their readiness for partnership and discern appropriate interventions that could enhance their readiness.

### Comparison with other studies

A comprehensive systematic review by Greenhalgh and collaborators identified 65 models and frameworks for patient engagement across 10 countries [21]. Notably, the UK (*n* = 34), United States (*n* = 14), and Canada (*n* = 7) emerged as frontrunners in the volume of published models and frameworks, highlighting their leadership in this field.

In the UK, the PPI initiative in clinical trials enhances patient engagement in research design. It has notably affected post-marketing clinical research, offering examples of successes and challenges in patient involvement across various stages [14,40,41]. A systematic review [41] indicated that PPI leads to study designs that better meet patient needs and improves participant experiences. However, challenges persist, including concerns about early involvement in the design process, accommodation of disabilities, resource availability, unclear roles, and the risk of tokenism. Researchers also grapple with maintaining rigor while addressing patient preferences and managing potential scope creep from personal priorities. Similar to the patient engagement frameworks currently being developed and implemented in the United States, interested parties perceive significant challenges in implementing the PPI framework despite its potential for improvement of trial design and the patient experience[44].

In the USA, the Patient-Centered Outcomes Research Institute (PCORI) emphasizes multi-party engagement, prioritizing the patient voice in all research phases [42,43]. While PCORI mainly focuses on real-world evidence rather than clinical trials, it has raised awareness of the importance of patient engagement by linking certain funding programs to it. The Science of Engagement initiative specifically funds studies on best practices for involving patients, caregivers, and community members in health research [42]. This initiative evaluates the quality and effectiveness of patient engagement, which may inform future research applications. PCORI outlines six core engagement principles to guide effective collaboration with patient partners, including reciprocal relationships, co-learning, valued partnerships, and transparent communication [42]. Although the DMD research partnership is not affiliated with PCORI, aspects of these principles, like co-learning, are evident in its collaborative culture. For practical guidance on engaging patient communities, PCORI offers the Engagement Rubric, covering the entire study lifecycle from planning to dissemination [42]. However, it lacks strategies for fostering long-term community partnerships. The Engagement Rubric primarily reflects strategies used in publically funded projects, which may not directly apply to industry-sponsored clinical trials. As the FOCUS model is adapted for new research partnerships, insights from the PCORI Science of Engagement Initiative may be useful.

In Canada, the Strategy for Patient-Oriented Research (SPOR), initiated by the Canadian Institutes of Health Research (CIHR), integrates patients into the research process [44]. Established in 2014, the SPOR Patient Engagement Framework outlines four core principles: inclusiveness, support, mutual respect, and co-build, aimed at fostering meaningful patient involvement in healthcare decisions [45]. While widely applied in research, clinical practice, and health policy [46–48], challenges such as power dynamics where patients have limited influence remain to be addressed for effective engagement [49].

Many published frameworks for patient engagement have seen limited use outside of the groups that created them [21]. The FOCUS model and blueprint is a standardized approach that can be used by other patient communities hoping to achieve an effective and enduring partnership with scientific, medical, and regulatory interested parties, like the partnerships the DMD community has forged with key players for more than 30 years [50]. The DMD community is unique in the clinical research landscape in their ability to recognize shortcomings in the early disease-modifying development programs for this condition and proactively partner with clinical researchers to address these shortcomings. To improve future clinical trials, they have collaborated with academic and industry researchers on numerous occasions to develop novel outcome measures, provide input on their lived experience to inform trial design, and formalize these improvements in an FDA guidance document that will provide a roadmap for DMD research for years to come.

### Strengths, limitations, and opportunities for future investigation

An important component of the FOCUS model was the identification of factors that contribute to successful partnerships. This was achieved through a study that was designed with the input of two key parties in the DMD research partnership, both of whom are advocacy leaders and one of whom is a parent of a person with DMD.

Forward translation and application of the FOCUS model will depend on validation of the model and appropriate selection and customization of interventions for specific target communities. All of these activities provide opportunities for further investigation and development that are outside the scope of the current project. Future work in this area may establish validity of the model, describe the customization or application of the model to individual communities through the use of complexity theory, or measure uptake of the model among the rare disease community as a whole.

This study’s particular scope and focus was limited to examination of the factors that have enabled the DMD community to establish and maintain a research partnership for drug development, and the study was grounded in an implementation science framework, the Behavior Change Wheel [35]. Other foundational perspectives or examination of additional research questions could be fruitful avenues for future investigation.

## Conclusion

Patient communities could apply the FOCUS model to design specific interventions tailored to their needs in order to establish or potentiate their partnerships with scientific, medical, and regulatory interested parties. In addition to validating the FOCUS model and exploring tailored interventions for specific communities, future researchers may wish to investigate strategies to maximize and evaluate the positive impact of patient engagement in the clinical research process. In the tradition of scientific inquiry, patient partnership remains imperfect. Through application of the FOCUS model, this study hopes to contribute to the improvement of the clinical research process for the benefit of the scientific enterprise and the patients it serves.

## Data Availability

The data that support the findings of this study are available from the corresponding author, SF, upon reasonable request.
